# Introduced Amino Terminal Epitopes Can Reduce Surface Expression of Neuronal Nicotinic Receptors

**DOI:** 10.1371/journal.pone.0151071

**Published:** 2016-03-10

**Authors:** John R. Bracamontes, Gustav Akk, Joe Henry Steinbach

**Affiliations:** 1 Department of Anesthesiology, Washington University School of Medicine, Saint Louis, MO, United States of America; 2 Taylor Family Institute for Innovative Psychiatric Research, Washington University School of Medicine, Saint Louis, MO, United States of America; Weizmann Institute of Science, ISRAEL

## Abstract

Epitopes accessible on the surface of intact cells are extremely valuable in studies of membrane proteins, allowing quantification and determination of the distribution of proteins as well as identification of cells expressing large numbers of proteins. However for many membrane proteins there are no suitable antibodies to native sequences, due to lack of availability, low affinity or lack of specificity. In these cases the use of an introduced epitope at specific sites in the protein of interest can often provide a suitable tool for studies. However, the introduction of the epitope sequence has the potential to affect protein expression, the assembly of multisubunit proteins or transport to the surface membrane. We find that surface expression of heteromeric neuronal nicotinic receptors containing the α4 and β4 subunits can be affected by introduced epitopes when inserted near the amino terminus of a subunit. The FLAG epitope greatly reduces surface expression when introduced into either α4 or β4 subunits, the V5 epitope has little effect when placed in either, while the Myc epitope reduces expression more when inserted into β4 than α4. These results indicate that the extreme amino terminal region is important for assembly of these receptors, and demonstrate that some widely used introduced epitopes may severely reduce surface expression.

## Introduction

Receptors for neurotransmitters mediate cellular responses to extracellular ligands and their known physiological role requires that they be expressed on the surface membrane of cells, often in particular regions (e.g. subsynaptic membrane). For this reason it is valuable to have probes for the presence of these receptors that recognize them in intact cells, in normal conditions. Some receptors have small molecule or toxin probes that associate with extracellular regions and can be used for this purpose, but antibodies to either native or introduced epitopes in the extracellular domain are the most widely used reagents. Introduced epitopes are commonly used when antibodies to native epitopes are unavailable, of low affinity on intact receptors, or demonstrate too high a level of cross-reactivity. There are a number of specific epitope sequences available, with well-characterized and relatively low-cost antibodies. However, the use of introduced sequences raises the possibility that expression of the mature receptor on the surface may be changed as a result of altered synthesis or folding of individual subunits, assembly of subunits or transport of the mature receptor to the cell surface.

Transmitter-gated membrane channels in the pentameric ligand-gated ion channel (PLGIC) family are multimeric proteins whose subunits must assemble in intracellular compartments and then the assembled receptors must be transported to the surface membrane to serve their physiological function. The subunits in this gene family share a common overall structure, with a large extracellular amino-terminal domain followed by three transmembrane domains. A relatively large intracellular loop occurs between the third and fourth transmembrane domains, and a short extracellular domain occurs at the carboxy-terminal. A series of experiments have demonstrated that successful surface expression can be disrupted by alterations in each of these regions [1], indicating the multiplicity of the interactions involved.

We have been studying members of this gene family, and use both native and introduced epitopes to quantitate the numbers of receptors on the cell surface. We and others have inserted epitopes into the amino-terminal region of several subunits of the GABA_A_ receptor, without significant effects on surface expression of receptors (HA [2], FLAG [3, 4], Myc [3, 4] epitopes, an α-bungarotoxin-binding motif [5] and even fluorescent proteins [5, 6] However, when we extended this work to the α4 neuronal nicotinic subunit we found that surface expression was reduced by insertion of the FLAG epitope. Insertion of a series of epitopes demonstrates that some epitopes greatly reduce surface expression while others have no significant effect. Studies of the β4 and β2 subunits indicate that this sensitivity to introduced sequences occurs in other neuronal nicotinic receptor subunits. Overall the data suggest that extended α-helical content at the extreme amino-terminus of these subunits may reduce their ability to assemble.

## Materials and Methods

### cDNA constructs and mutagenesis

Human α4, β2, and β4 cDNAs were obtained from Dr. Lindstrom (University of Pennsylvania, Philadelphia, PA). Each of these cDNAs was transferred to the pcDNA3 expression vector (Life Technologies, Grand Island, NY), and various epitope tags and sequences were introduced through mutagenesis. Each of the three cDNA constructs was mutated to include the tags or sequences near the N terminus of the mature peptide. The QuikChange (Agilent Technologies, Santa Clara, CA) mutagenesis method was used to introduce the indicated modifications. Full-length sequencing of the coding region was done to validate the mutations and to show that no other changes were made.

The constructs, from the predicted amino terminus, had the sequences (inserted sequence underlined):

Human α4:

FLAG4: HVETDYKDDDDKRAH

FLAG9: HVETRAHAEDYKDDDDKERLL

Myc: HVETEQKLISEEDLRAH

HA: HVETYPYDVPDYARAH

V5: HVETGKPIPNPLLGSLDTRAH

PolyA: HVETAAAAAAAARAH

RC: HVETYKNWSPIPRAH

RC2: HVETRAHAEERLLKKLFSYKNLSPIPGYN

Human β4:

FLAG4: RVANDYKDDDDKAEE

Myc: RVANEQKLISEEDLAEE

V5: RVANGKPIPNPLLGSLDTAEE

RC: RVANYKNWSPIPAEE

RC2: RVANAEEKLMDDLLNYKNLSPIPKTR

Myc+T RVANEQKLISEEDLTAEE

RC+T: RVANYKNWSPIPTAEE

Human β2:

FLAG4: GTDTDYKDDDDKEER

V5: GTDTGKPIPNPLLGSLDTEER

### Cell culture, transfection, and enzyme-linked immunosorbent assay

Cell culture conditions, using human embryonic kidney (HEK) 293 cells, have been described in detail previously [7, 8]. Transfections were done using the Effectene transfection reagent (Qiagen, Valencia, CA) and cDNAs at a ratio of 1:1 α4:β2 or α4:β4. HEK 293 cells (ATCC, Gaithersburg, MD) were plated at a density of about 50,000 cells/well in 24-well dishes treated with poly-d-lysine (Biocoat, Horsham, PA). Transfections were done 1 day after plating, and enzyme-linked immunosorbent assays (ELISA) were performed 2 days after transfection.

The QT6 quail fibroblast cell line was cultured and transfected as described previously [9]. Cells were plated at a density of about 50,000 cells/well in 24-well dishes treated with poly-d-lysine (Biocoat, Horsham, PA). Transfections were done using the Effectene transfection reagent (Qiagen) at a ratio of 1:1 α4:β4. Transfections were done 1 day after plating, and enzyme-linked immunosorbent assays (ELISA) were performed 2 days after transfection.

ELISA assays were performed as previously reported [10]. In each experiment, for each set of subunits transfected, three wells were used for ELISA assays and two wells for a protein assay using a bicinchoninic acid method (Thermo Fisher Scientific, Waltham, MA).

The surface expression of the α4 subunit was assayed by the binding of a monoclonal antibody recognizing an extracellular epitope (mAb 299; Sigma-Aldrich, St. Louis, MO), and a horseradish peroxidase–coupled IgG anti-rat antibody (GE Healthcare Bio-Sciences, Piscataway, NJ). Surface expression of the FLAG epitope was assayed by the binding of the anti-FLAG monoclonal antibody (m2; Sigma-Aldrich, St. Louis, MO), and a horseradish peroxidase IgG anti-mouse antibody (GE Healthcare Bio-Sciences). Cells were washed in phosphate-buffered saline (PBS) (137 mM NaCl, 2.7 mM KCl, 4.3 mM Na_2_HPO4, and 1.4 mM KH_2_PO4, pH 7.3), blocked with 4% (w/v) powdered milk in PBS for 30 minutes at room temperature, and then incubated with primary antibody for 60 minutes at room temperature. After a wash with milk-PBS, the cells were incubated with horseradish peroxidase conjugated secondary antibody (60 minutes), washed again with milk-PBS four times then washed with PBS four times, and finally incubated with the horseradish peroxidase substrate 1-Step Ultra TMB (3,3′,5,5′ tetramethylbenzidine; Thermo Fisher Scientific). The absorbance of the supernatant was measured with a plate reader (iMark; Bio-Rad, Hercules, CA

ELISA results were analyzed by subtracting machine background, then normalizing the ELISA optical density signal to the cell protein. We then subtracted the mean normalized signal from the negative control from all values. To control for variability in transfection, all the data were then normalized to the relative ELISA for the positive control obtained for that transfection. The end result is a number giving the expression relative to that for wild-type receptors, where 1 means equivalent expression and 0 expression equivalent to the negative control.

### Western blotting

Western blotting was performed on extracts from transiently transfected HEK cells. A 150mm cell culture dish was seeded with cells at about 50% confluency. Transfection was done the following day using Effectene. Cells were harvested the second day after transfection. Cells were washed 1x with 20 ml ice cold PBS followed by a second wash with 10 ml ice cold PBS. A third wash with 5 ml of PBS with protease inhibitors (P8465; Sigma-Aldrich, St. Louis, MO) was done before scraping of cells off the dish. Cells were scraped from the dish after adding 2 ml of PBS + inhibitors, and cells were subsequently transferred to a 15 ml centrifuge tube. The plate was rinsed with another 2 ml of PBS + inhibitors and added to the centrifuge tube. Cells were centrifuged at 800 rpm for 5 min., 4°C. Supernatant was removed and 1ml PBS(300mM NaCl)/inhibitors was added to cells which were then transferred to a 1.5ml microcentrifuge tube. Cells were lysed with two freeze thaw cycles in liquid nitrogen. Lysate was centrifuged and supernatant was discarded, retaining the pellet. The membrane pellet was resuspended with PBS + inhibitors + 2% triton X-100 and extracted at 4°C 1 hour. Extracted lysate was centrifuged 5 min at 1000 rpm to remove cell debris. Supernatant was retained for PAGE and Western blot.

An equal volume of 2x Laemmli buffer was added to the protein extracts and incubated at room temperature for one hour. Fifty microliters (25 μg total protein) of each sample were then loaded onto a precast 4 to 15% gradient Tris-glycine polyacrylamide gel (Bio-Rad) and electrophoresed. The gel was then transferred to a nitrocellulose Hybond-ECL membrane (GE Healthcare, Chalfont St. Giles, Buckinghamshire, UK). The membrane was preblocked in 100% Odyssey block solution (LI-COR Biosciences, Lincoln, NE) at room temperature for 1 h, followed by overnight incubation in a solution of 50% Odyssey block solution: 50% phosphate-buffered saline (PBS; 137 mM NaCl, 2.7 mM KCl, 4.3 mM Na2HPO4, and 1.4 mM KH2PO4, pH 7.3) containing 0.2% Tween 20 (Thermo Fisher Scientific, Waltham, MA) with primary antibody. The primary antibody was raised to the cytoplasmic loop of the α4 subunit (H-133, 1:500 dilution; Santa Cruz Biotechnology, Santa Cruz, CA). The membrane was washed with PBS + 0.2% Tween 20, nine times, then incubated with goat anti-rabbit IRDye 680 (LI-COR Biosciences) at a 1:2000 dilution in a solution of 50% blocking buffer and 50% PBS with 0.2% Tween 20 at room temperature for 30 min. The membrane was washed as before followed by a rinse in PBS. Bands were visualized using the Odyssey system (LI-COR Biosciences).

### Electrophysiology

Voltage-clamp recordings were made from transfected HEK cells 2 to 3 days after transfection, as described previously [11]. Cells expressing a high density of surface receptors were identified with a bead-labeling technique [9], using mAb299 to provide a qualitative measure of the surface level of the α4 subunit. In previous work we have found that many cells show no response to acetylcholine, presumably because of an absence of successful transfection or expression [12, 13]. The response to a high concentration of acetylcholine (1 mM) was used to indicate the functional status of the receptors and the response to a lower concentration (10 μM) was used to determine whether large differences in the potency of acetylcholine were present.

### Predictions of secondary structure

We used programs available on the World Wide Web to predict changes in secondary structure as a result of the inserted sequences, in all cases using the default parameters. The region analyzed was the initial amino-terminal portion of the predicted mature subunit (see Results). We used GORV (http://gor.bb.iastate.edu/) and Jpred4 (http://www.compbio.dundee.ac.uk/jpred/) as well as the consensus prediction program Sympred (http://www.ibi.vu.nl/programs/sympredwww/). In the case of Sympred, the default prediction programs used to generate the consensus were PROFsec, SSPro 2.01, YASPIN and PSIPred. As noted in Results, the predictions were relatively similar for some sequences (e.g. for wild-type α4 and β4 subunits) but diverged for some inserted sequences.

### Data analysis

Results are presented as mean ± SE (number of experiments). Statistical tests on surface ELISA results relative to wild type subunits were performed by ANOVA with Dunnett’s correction for multiple comparisons (STATA, StataCorp, College Station, TX); the tests were performed separately for comparing the α4 and β4 subunits. In some cases t-tests (unpaired, unequal variances) were performed to compare two sets of data (e.g. α4β2 vs. α4β4) (Excel, Microsoft Corporation, Redmond, WA). Correlation between surface ELISA and predicted structural features was examined using Excel.

## Results

### Epitope insertion into the α4 subunit can reduce surface expression of α4β4 receptors

**Fig 1:**
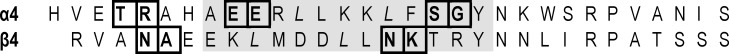
Sequences of the amino-terminal regions of the subunits studied. The sequences of the mature subunit from the predicted amino-termini are shown for the human nicotinic α4 and β4 subunits are shown. The locations of insertions are shown by the boxed residues (e.g. insertions were made between α4 T4 and R5). The region indicated by gray shading is likely to be α-helical based on alignments to related subunits of known structure (see Text for details). The two leucine residues shown in *italics* are residues identified as important for expression in the nicotinic α7 subunit (see Text).

**Fig 2:**
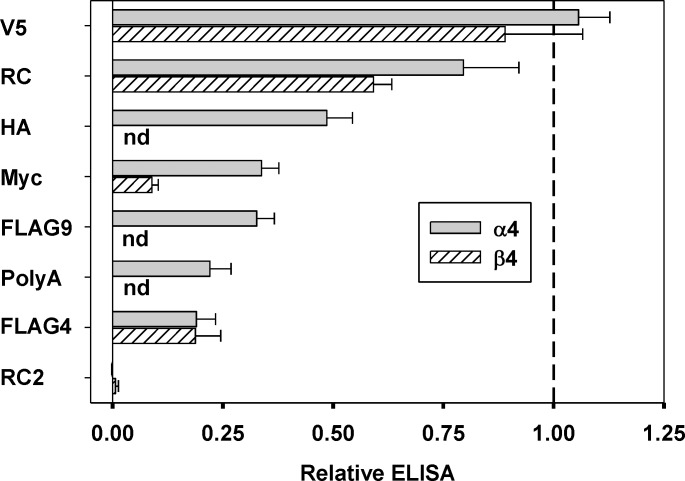
Comparison of relative surface expression for different constructs. The same sequence was inserted into the α4 and β4 subunits, with generally similar effects on surface epitope expression. The only pair that differed significantly in relative surface expression was when the Myc epitope was inserted (P = 0.005 that the difference would have arisen by chance). Data shown are mean ± SE; for numbers of transfections see Table 1. The dashed vertical line shows a value of 1, corresponding to the expression seen for wild-type α4 and β4 subunits, and the thin vertical line shows a value of 0, corresponding to the expression seen in the negative control (pcDNA3 vector). “nd” indicates that the construct was not studied.

We and others have inserted the FLAG epitope (DYKDDDDK) near the amino-terminal end of GABA_A_ receptor subunits without reducing surface expression [3, 4]. Accordingly, we constructed a nicotinic α4 subunit with FLAG inserted at a homologous position between residues 4 and 5 of the mature subunit (FLAG4; see Fig 1). We were surprised when initial results showed low binding of a monoclonal antibody to the FLAG epitope to cells expressing nicotinic receptors containing this subunit coexpressed with the nicotinic β4 subunit. We then used a monoclonal antibody to the α4 subunit that recognizes an extracellular epitope (mAb 299; [14]) to examine the level of surface expression in ELISA assays on intact cells. The data shown in Fig 2 and Table 1 demonstrate that the presence of the FLAG epitope significantly reduces the amount of α4 subunit that reaches the cell surface.

**Table 1:** Summary of expression data.

Insert	insertion in α4	insertion in β4
wt	1.00 (21)	1.00 (21)
V5	1.06 ± 0.07 (5) [0.81]	0.89 ± 0.18 (5) [0.96, 0.42]
RC	0.80 ± 0.13 (3) [0.013]	0.59 ± 0.04 (3) [0.15, 0.24]
HA	0.49 ± 0.06 (4) [<0.001]	nd
Myc	0.34 ± 0.04 (4) [<0.001]	0.09 ± 0.01 (3) [<0.001, 0.005]
FLAG9	0.33 ± 0.04 (4) [<0.001]	nd
PolyA	0.22 ± 0.05 (2) [<0.001]	nd
FLAG4	0.19 ± 0.04 (11) [<0.001]	0.19 ± 0.06 (5) [<0.001, 0.98]
RC2	0.00 ± 0.00 (3) [<0.001]	0.01 ± 0.01 (4) [<0.001, 0.41]
Myc+T	nd	1.20 ± 0.24 (6) [0.098,—]
RC+T	nd	1.45 ± 0.33 (6) [<0.001,—]

The first column identifies the construct in terms of the inserted sequence. The relative surface epitope expression when the insertion is made in the α4 subunit is shown in the second column, and expression when the insertion is made in the β4 subunit in the third column. Data are given as mean ± SE (number of transfections). Results of statistical tests are shown in the square brackets as the probability that the difference to the wild-type subunit would arise by chance (both columns; t-test with Dunnett’s correction for multiple comparisons) followed by the probability that the difference between results when the sequence is inserted in the β4 subunit versus the α4 subunit would arise by chance (only in β4 column; unpaired t-test with unequal variance). “nd” indicates that the construct was not studied.

To explore this further, we inserted the Myc (EQKLISEEDL), HA (YPYDVPDYA) and V5 (GKPIPNPLLGLDST) epitope tags at the same position and assayed surface levels of α4 using mAb 299. As shown in Fig 2 and Table 1 the different epitopes had differential effects on surface expression: FLAG4 produced the greatest reduction (to 19% of wild type), HA and Myc produced partial reduction (to 49% and 34%, respectively) while V5 had no significant effect. Clearly the nature of the epitope can influence the effect on expression.

In the absence of a β subunit, surface expression of α4 was not significantly different from the negative control (empty pcDNA3): for α4 subunits expressed in the absence of any β subunit the binding of mAb299 was 0.010 ± 0.007 (N = 2) relative to binding when α4 was expressed with β4.

**Fig 3:**
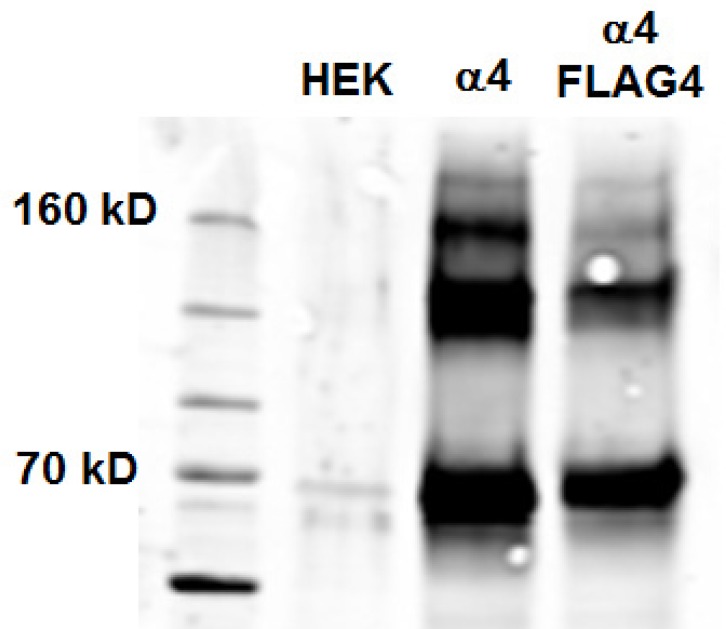
Immunoblot comparing total expression of α4 immunoreactive material between α4β4 receptors and α4FLAG4β4 receptors. The figure shows a molecular weight ladder (1st lane), then protein from untransfected HEK cells (“HEK”), from cells transfected with α4 and β4 subunits (“α4”) and from cells transfected with α4FLAG4 and β4 subunits (“α4FLAG4”). The α4 subunit was detected using an antibody against the cytoplasmic loop. Quantitation of the bands at about 70 kD reveals a modest decrease in the α4 FLAG β4 receptor compared to wild type (to 74% of wild-type). The α4 subunit is expected to migrate with an apparent molecular weight of about 67 kD. The more slowly migrating, likely aggregated, material showed a similar reduction, to 73% of wild type.

To determine whether insertion of the FLAG epitope affected the total level of synthesized subunits, we performed Western blots of total protein using an antibody to an epitope in the major cytoplasmic loop of the α4 subunit (Fig 3). In two separate experiments the total level of α4FLAG4 was reduced to 53% and 74% of the wild-type level. This reduction is less than the reduction in surface protein (to 19%) suggesting that the major effect on surface expression is not reduced synthesis or accumulation of α4 protein, but instead reflects deficits in maturation or assembly of receptors, or transport to the surface.

### Epitope insertion into a β subunit can reduce surface expression of α4-containing receptors

We then made analogous insertions in the β4 subunit (Fig 1). The FLAG epitope again reduced surface expression to 20% of wild type, while Myc reduced expression to a greater extent and V5 did not significantly affect expression (Fig 2, Table 1). In comparing the extent of surface expression when an epitope was placed in α4 versus β4, FLAG4 and V5 had similar effects in either subunit (P = 0.97 for FLAG4 and 0.58 for V5 that the differences between the levels when the insertion was made in the α4 versus β4 subunit were due only to chance; unpaired t-test). For Myc the level of expression when the epitope was placed in the β4 subunit was lower than when in α4 (P = 0.005).

Clearly, insertion of an epitope into either the α4 or β4 subunit can reduce expression, although there may be some difference in the extent of expression depending on the subunit and introduced sequence.

The α4 subunit is often expressed in receptors that also contain the β2 subunit, so we tested whether expression of receptors containing the β2 subunit was also sensitive to insertion of an epitope. Surface expression of the receptors when the α4 subunit was expressed with the wild-type β2 subunit was reduced compared to expression with the β4 subunit (relative expression 0.33 ± 0.03, N = 3; P = 0.002), as also noted earlier [15]). Insertion of FLAG into the β2 subunit between the fourth and fifth residues reduced mAb 299 binding to background levels (relative expression 0.000 ± 0.002, N = 3) while insertion of V5 reduced expression to 0.44 ± 0.07 (N = 3) of the level seen with wild type β2 (in both cases P < 0.001 for difference to wild-type β2). Accordingly, the β2 subunit shows qualitatively comparable effects of epitope insertion as the α4 and β4 subunit.

Expression of β4V5 alone was tested using antibody to V5; surface expression of β4V5 alone was 0.012 ± 0.007 (N = 3) relative to β4V5 expressed with α4. Accordingly, the vast majority of surface receptors contain both α4 and β subunits.

### Receptors with V5 epitope insertions are functional

**Fig 4:**
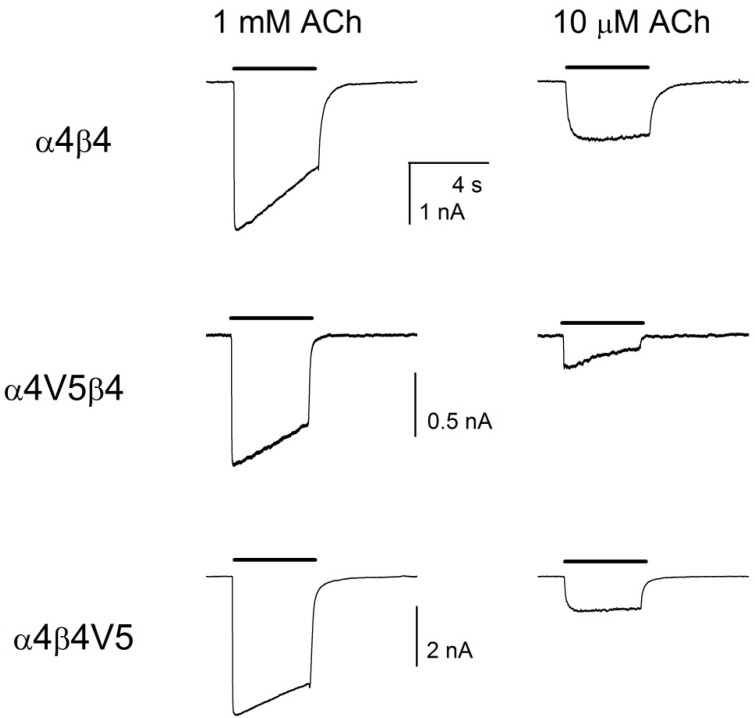
Receptors containing subunits with the V5 epitope are functional. The figure shows responses of cells to applications of acetylcholine (left column 1 mM, right column 10 μM). Each row shows responses from a single cell to the application of different concentrations of acetylcholine (top row wild-type subunits, middle row α4V5β4, bottom row α4β4V5). The horizontal scale bar shows 4 sec for all panels, the vertical scale bar in the left column shows the current calibration for both panels in that row.

We recorded whole-cell currents evoked by acetylcholine from receptors composed of wild-type α4 with β4 subunits, α4V5 with β4, and α4 with β4V5. As shown in Fig 4 all 3 types of receptors resulted in robust evoked responses. Cells were selected for study using beads and monoclonal antibody 299 (see Methods) to identify cells that expressed surface receptors. The amplitudes of responses to 1 mM acetylcholine were: α4β4 1468 ± 640 nA (6 cells; mean ± SE), α4V5β4 608 ± 92 nA (6) and α4β4V5 2650 ± 740 nA (5), which did not differ significantly (P > 0.06 for any pairwise comparison, one way ANOVA with Bonferroni correction). The relative responses to 10 μM acetylcholine were: α4β4 0.38 ± 0.09 (3), α4V5β4 0.23 ± 0.02 (4) and α4β4V5 0.24 ± 0.02 (4), which also did not differ significantly (P > 0.2). Responses showed some decrease during the 5 sec application of 1 mM acetylcholine, likely due to receptor desensitization, but the amount varied between cells and did not differ significantly between receptors of different composition (P > 0.1). We did not characterize the functional properties further.

### Insertion of epitopes has similar effects on expression in quail fibroblasts

We expressed some combinations of subunits in the QT6 quail fibroblast cell line [9], to determine whether the inserted epitopes had different consequences in other expression systems. When compared to wild-type subunits, expression was reduced when α4FLAG4 was expressed with β4 (the mean relative expression was 0.20; 2 independent values 0.09 & 0.32) but not when α4V5 was (1.15; 1.25 & 1.04). Similarly, β4FLAG reduced surface expression (0.24; 0.24 & 0.24) to a lesser extent than β4V5 (0.66; 0.73 & 0.59). Accordingly the effects of the insertions are not restricted to the HEK expression system.

### Effects of other insertions

**Fig 5:**
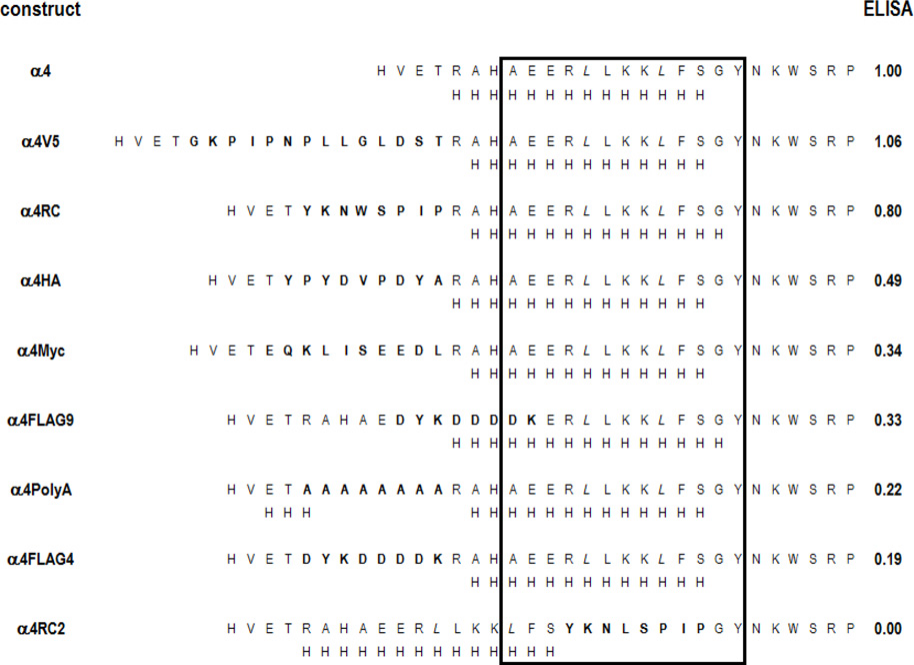
Sequences and predicted structures of the α4 constructs studied. The first column identifies the construct. Then the N-terminal amino acid sequence is shown aligned at the conserved RP motif, with the insert shown in bold letters. Immediately below the sequence is the secondary structure predicted by Sympred (see Methods). The last column shows the mean relative ELISA signal. The boxed region is likely to be helical based on alignments to related subunits of known structure.

**Fig 6:**
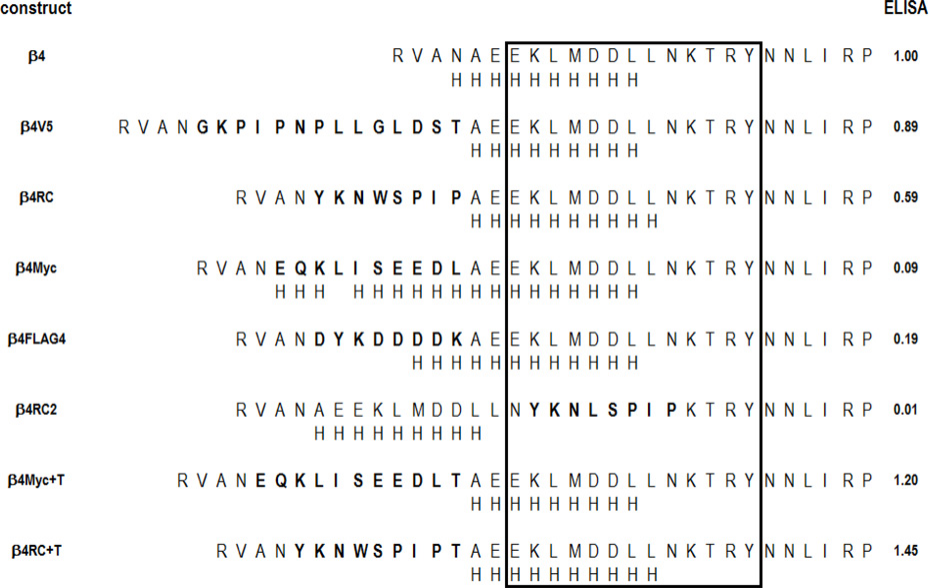
Sequences and predicted structures of the β4 constructs studied. The amino acid sequences are shown as in Fig 5.

We tested two additional inserted sequences that are not epitope tags (Figs 5 and 6). One is a sequence of 8 alanines (“PolyA”) which is likely to enhance an α-helical structure. The second is the sequence YKNWSPIP (“RC”), predicted to adopt a random coil structure. As shown in Table 1, insertion of PolyA into α4 reduced surface expression to about 20%, whereas insertion of RC reduced it to only 80%. Insertion of RC into β4 produced a comparable reduction in insertion (Table 1).

Finally, we fortuitously tested two epitope tags with an additional threonine residue added at the carboxyl terminal end of the tag, Myc+T and RC+T, each inserted between residues 4 and 5 of the β4 subunit (sequences shown in Fig 6). Both of these constructs expressed at higher levels than the tag without the additional T (Table 1). In the case of Myc+T the difference was significant (P = 0.006) while with RC+T the difference was marginal (P = 0.048).

Structural studies of muscle nicotinic receptors (Torpedo, [16, 17]; mouse α1 subunit [18]) and related receptors and proteins (glutamate activated chloride channel (GluCl; [19]), GABA_A_ β3 subunit [20], acetylcholine binding protein (AChBP [21]), serotonin type3 α subunit [22]) show an α helix near the amino terminal end (termed the “α1 helix”), and prediction programs also predict that the nicotinic α4 and β4 subunits contain such a region (Figs 5 and 6). Previous studies of the nicotinic α7 subunit have concluded that the α1 helix plays an important role in assembly of this subunit into pentameric receptors [23]. All of the insertions we have described so far were placed before the start of the α1 helix, based on alignments with the sequences for which there is structural information (Figs 1, 5 and 6). We also made 3 insertions into the predicted helix. We placed the FLAG epitope into the amino terminal helix of α4 (between residues 9 and 10 of the mature subunit; FLAG9), which resulted in expression at 33% of wild type, slightly more than seen when FLAG was placed between residues 4 and 5. We also placed a sequence predicted to form a random coil (RC2, YKNLSPIP) into the helix, between residues 18 and 19 in α4 and residues 15 and 16 in β4. In both cases surface expression was reduced to background levels.

### Effects of insertions on predicted subunit structure

From these results it can be seen that insertion of various sequences in this region can result in a variety of effects on surface expression, from complete suppression to no effect or even an increase of surface levels. Inspection of the introduced sequences (Figs 5 and 6) suggests that the specific amino acids are not the critical factor. For example, in the α4 subunit the FLAG4 and PolyA sequences had similar effects on surface expression but differ greatly in the nature of the introduced residues. On the other hand, the RC and RC+T insertions into the β4 subunit are very similar in amino acid sequence, yet had very different consequences for surface expression.

Accordingly, we examined the predicted changes in secondary structure. Because the studies of the α7 subunit showed major consequences for mutations in the α1 helix itself, we analyzed the predicted changes in structure for the region starting at the predicted amino terminus and extending to the conserved arginine-proline (RP) pair (see Figs 5 and 6). We used a subset of programs available on the World Wide Web and analyzed a “consensus” prediction produced by Sympred (a total of 6 programs, see Methods). The programs agreed fairly well in predicting the structure of wild-type α4 and β4, but some of the predictions for subunits with introduced sequences diverged between programs (S1 Fig). Accordingly, the structure in this region may not be well defined.

**Fig 7:**
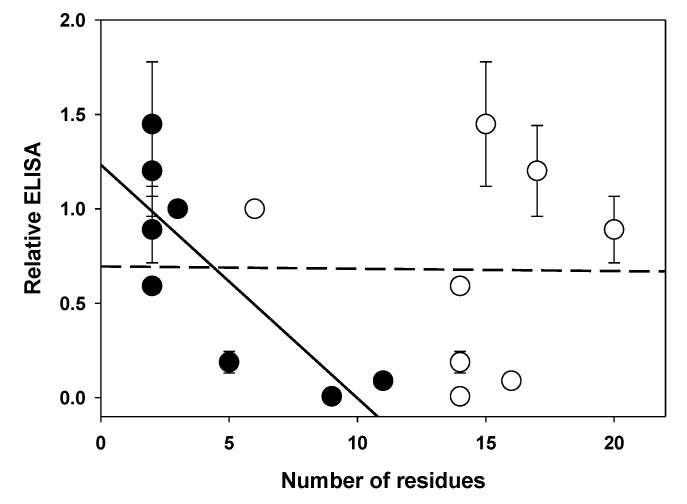
Relationship between relative ELISA signal and predicted structural features in the β4 subunit. The mean ELISA signal is plotted against the total number of residues in the N-terminal region preceding the predicted α1 helix (hollow circles) and the number of residues in this region predicted to be in an α helix (solid circles). The lines show linear regressions (dashed–total number, slope = -0.001, probability it differs from a slope of zero P = 0.43; solid–helical number, -0.12 P = 0.01). Points show mean ± SE (for values see Table 1); wild-type subunits have a relative ELISA value of 1.

We extracted several relatively crude parameters from the predictions, and then examined correlation between the relative surface ELISA signal and the parameter. There were no significant correlations between the relative ELISA signal and the total length of the sequence preceding the α1 helix, the number of residues predicted to be helical in the putative α1 helix or the total number of residues predicted to be in helical regions for the entire sequence analyzed, for either subunit (Pearson’s product moment correlation r values between -0.50 and 0.54; P > 0.16 the value for r was indistinguishable from 0 for all correlations). The only parameter that correlated with surface ELISA was the number of residues predicted to be in an α helix in the sequence preceding the α1 helix, for which an increased number of helical residues was correlated with a decreased ELISA (Fig 7). The association was relatively weak (r = -0.56, P = 0.12 for α4, -0.81, P = 0.01 for β4) given the number of correlations examined. This observation might suggest that increased α-helical content before the α1 helix resulted in a negative effect on assembly.

This suggestion receives support from a comparison of the effects of β4Myc to β4Myc+T, or between the effects of Myc when introduced in the β4 and the α4 subunit (Figs 5 and 6). In each pair an increase in the predicted extent of the N-terminal helical region is associated with reduced surface expression.

## Discussion

These results demonstrate that inserted epitope sequences can have major effects on the expression of neuronal nicotinic receptors that depend on the nature of the sequence inserted. This is perhaps not surprising, although it clearly demonstrates the difference between these nicotinic subunits and related GABA_A_ receptor subunits including α, β and γ subunits. Using a number of different GABA_A_ receptor subunits, a number of experimenters have inserted HA [2], FLAG [3, 4], Myc [3, 4] epitopes, an α-bungarotoxin-binding motif [5] and even fluorescent proteins [5, 6] in this region of the protein with minimal consequences for expression. Among the epitopes we tested, the V5 sequence appeared to be the best tolerated by neuronal nicotinic subunits.

The α4 and β4 subunits can assemble in (at least) 2 stoichiometries: 3 copies of α4 and 2 of β4, or 2 of α4 and 3 of β4 [24], as is true for other combinations of neuronal nicotinic α and β subunits [25, 26]. This could potentially affect the present results: if the stoichiometry were changed from 100% of the type containing 3 α4 to 100% of the type containing 2 α4 there would be a 33% reduction in surface expression of α4 even with no change in surface receptor number, while the converse change would result in a 50% increase. We did not directly examine the subunit stoichiometry of the surface receptors. However, one assay is the concentration of acetylcholine producing a half-maximal effect (the EC_50_) that is typically lower for receptors containing 3 copies of a β subunit [24–26]. We did not measure the EC_50_, but found that the relative response to 10 μM acetylcholine did not differ between receptors containing wild-type subunits or subunits with the V5 epitope inserted. This suggests that the subunit composition did not differ, at least among these receptors. In addition, most of the statistically significant effects shown in Table 1 indicate larger reductions than 33%. It is also notable that the insertions in α4 and β4 have parallel effects on surface levels of α4. This would be unlikely if the insertions had similar consequences for assembly of the two subunits, as a reduction in β4 incorporation into a constant number of surface receptors might be expected to enhance the surface exposure of α4 and *vice versa*. Accordingly, we cannot rule out the possibility that changes in subunit stoichiometry contribute to the results we obtained, but they seem unlikely to account for the major effects seen. It would be very interesting if there were systematic changes in stoichiometry and the possibility clearly deserves further study.

Much of the previous study of the role of the amino-terminal regions in surface expression has involved deletion of regions. In the case of the ρ1 GABA_C_ subunit, deletion of the amino-terminal extension preceding the α1 helix reduced surface expression, and deletion of both the extension and the α1 helix abolished surface expression [27]. Similarly, a study of the GABA_A_ α1, β2 and γ2 subunits found that removal of the N-terminal extension greatly reduced assembly of receptors when the deletion was made the in α1 subunit but not in the β2 or γ2 subunits [28]. Further deletion of the α1 helices in β2 or γ2 did decrease surface expression. These studies demonstrate that major changes in the amino-terminal regions can strongly affect assembly of subunits, and that the effects are dependent on the particular subunit studied. The authors emphasize that the N-terminal extension can play an essential role in promoting assembly of pentameric receptors in the PLGIC gene family. These studies, particularly of the GABA_A_ β2 and γ2 subunits, suggest that the region is relatively insensitive to the nature of the sequence located in the N-terminal extension, but successful assembly appears to require some (perhaps unstructured) peptide sequence.

In contrast, our constructs involved insertions of sequences into the extreme N-terminal regions of the subunits. Our experiments did not resolve a structural basis for the effects, although there was a suggestion that increased extension of the α1 helix towards the amino-terminus is associated with lower expression. There was not any correlation with other predicted structural changes in the structure of the region, and no clear association with particular amino acid residues.

It has been proposed that residues in the α1 helix of the nicotinic α7 subunit interact specifically with residues in other portions of the extracellular domain [29]). An extensive series of chimeric constructs involving replacement of the α1 helix with homologous helices from other subunits in the PLGIC family demonstrated that helices from the nicotinic α3, α5 and β3 subunits reduced surface expression. The effect on surface expression was, in turn, reduced when sequence of another region of the relevant subunit was also transferred to the α7 extracellular domain (e.g. both the α1 helix and the β2-β3 loop regions from the α3 subunit). These results provide strong evidence that it is not simply the presence of any α1 helix that is important, but the presence of a particular helix that is likely to interact with other regions of the subunit. In the case of the GABA_A_ β3 subunit a mutation of a residue immediately before the predicted α1 helix [30] reduces surface expression, and based on position in a homology model it was suggested that the mutation affected interactions with an adjacent subunit. Both of these studies concerned the properties of the α1 helix rather than the N-terminal extension, but do indicate that some portions of the extreme N-terminal region form specific interactions with other portions of the receptor.

In our studies the only insertion that significantly affected the structure of the α1 helix was the RC2 construct, which abolished surface expression. We note that previous work [29] had found that mutation to proline of either conserved leucine in the predicted α1 helix of the nicotinic β4 subunit (sequences in S2 Fig) essentially abolished surface expression of receptors containing the α3 and β4 subunits with little change in the predicted α1 helix (S2 Fig). Our RC2 constructs changed the position of these residues in the assembled receptor and so might have disrupted interactions. In addition, this insertion moved the predicted helical region towards the amino-terminus.

It should be noted that studies of the α7 homopentameric receptor may be complicated by changes in the affinity of the probe (in this case α-bungarotoxin). A study of a chimeric subunit comprising the chicken α7 extracellular domain (from the N-terminus to the first membrane-spanning helix) joined to the membrane spanning and major intracellular portions (the start of the first membrane-spanning helix to the C-terminus) of the *C*. *elegans* GluCl receptor provided some additional information [31]. It was found that deletion of the region from the N-terminus through the α1 helix did indeed reduce surface binding of α-bungarotoxin to background levels and no response to acetylcholine or nicotine was recorded from transfected cells. However, when an HA tag was incorporated at the amino-terminal end of the subunit surface expression of the HA epitope was detected and Western blots indicated that the subunit could assemble into high molecular weight forms even after deletion of the α1 helix. These latter observations suggest that assembly and transport of receptors occurred, but the receptors were compromised both functionally and in terms of probe affinity. Accordingly, the overall interpretation of the studies of the α7 subunit is not completely settled.

One difficulty in identifying the relationship between structure in this region and receptor expression is that the amino-terminal sequence preceding the α1 helix is not resolved in most of the structures determined so far. Several proteins have a minimal N-terminal sequence preceding the α1 helix (the nicotinic α1 subunit and AChBP), while the sequence has been deleted or shortened in other proteins used for crystallization (GluCl and 5HT3). The amino-terminal sequence could be resolved in only one subunit in a pentamer of GABA_A_ β3 subunits [20]; in this case it appears to bend back towards the main part of the extracellular domain of the same subunit.

In sum, the present results confirm that the first 20 or so residues of neuronal nicotinic receptor subunits can have a major effect on surface expression of receptors. Furthermore, they demonstrate that inserted sequences as well as deletions, point mutations or chimeric constructs may greatly reduce surface expression. Our results indicate that the effects are qualitatively similar for 3 subunits (α4, β2 and β4), so this may be a general property of neuronal nicotinic subunits. The α and β subunits occupy different positions in the assembled pentameric receptor, suggesting that the reduction is not the result of altered specific interactions between residues in adjacent subunits. It seems possible that specific interactions among amino acid residues in a subunit or a steric effect on intersubunit packing underlie the effects. Inspection of the introduced sequences does not immediately suggest a pattern of the specific residues introduced, suggesting that a steric effect might be more likely. Finally, the results demonstrate that some widely used introduced epitopes may severely reduce surface expression of some neuronal nicotinic receptors, indicating that appropriate selection of an introduced epitope is critical.

## Supporting Information

S1 Fig Sequences and predicted secondary structures for selected constructs. (PDF)

S2 Fig Sequences and predicted secondary structures for 2 mutations in the α1 helix mentioned in the Discussion. (PDF)
