# Supplementary material for: Introduced Amino Terminal Epitopes Can Reduce Surface Expression of Neuronal Nicotinic Receptors
Source: PLoS One. 2016 Mar 10;11(3):e0151071. doi: 10.1371/journal.pone.0151071 (PMC4786271; doi:10.1371/journal.pone.0151071)
Supplement: S1 Fig — (PDF) [file pone.0151071.s001.pdf]

Bracamontes et al

Supplemental figure 1

| Construct | Pred | | | | | | | | | | | | | | | | | | | | | | | | | | | | | | | | | | | | | | | |
|---|---|---|---|---|---|---|---|---|---|---|---|---|---|---|---|---|---|---|---|---|---|---|---|---|---|---|---|---|---|---|---|---|---|---|---|---|---|---|---|---|
| α4 | AA | | | | | | | | | H | V | E | T | R | A | H | A | E | E | R | *L* | L | K | K | *L* | F | S | G | Y | N | K | W | S | R | P | V | A | N | I | S |
| α4 | GorV | | | | | | | | | | | | | | H | H | H | H | H | H | H | H | H | H | H | H | | | | | | | | | | | | | | |
| α4 | Jpred | | | | | | | | | | | | | | | H | H | H | H | H | H | H | H | H | H | H | H | | | | | | | | | | | | | |
| α4 | PROF | | | | | | | | | | | | | H | H | H | H | H | H | H | H | H | H | H | H | H | | | | | | | | | | | | | | |
| α4 | PSIPRED | | | | | | | | | | | | | H | H | H | H | H | H | H | H | H | H | H | H | H | H | | H | H | H | H | | | | | | | | |
| α4 | SSPRO | | | | | | | | | | | | | H | H | H | H | H | H | H | H | H | H | H | H | H | H | | | | | | E | | | | | | | |
| α4 | YASPIN | | | | | | | | | | | | | H | H | H | H | H | H | H | H | H | H | H | H | H | H | H | | | | | | | | | | | | |
| α4 | SYMPRED | | | | | | | | | | | | | H | H | H | H | H | H | H | H | H | H | H | H | H | H | | | | | | | | | | | | | |
| β4 | AA | | | | | | | | | | R | V | A | N | A | E | E | K | *L* | M | D | D | *L* | L | N | K | T | R | Y | N | N | L | I | R | P | A | T | S | S | S |
| β4 | GorV | | | | | | | | | | | | | H | H | H | H | H | H | H | H | H | H | H | H | H | | | | | | | | | | | | | | |
| β4 | Jpred | | | | | | | | | | | | | | H | H | H | H | H | H | H | H | H | H | | | | | | | | | | | | | | | | |
| β4 | PROF | | | | | | | | | | | | | H | H | H | H | H | H | H | H | H | H | | | | H | H | H | H | H | | | | | | | | | |
| β4 | PSIPRED | | | | | | | | | | | | | H | H | H | H | H | H | H | H | H | H | H | H | H | H | H | H | H | H | H | | | | | | | | |
| β4 | SSPRO | | | | | | | | | | | | | H | H | H | H | H | H | H | H | H | H | | | | | | | | | | | | | | | | | |
| β4 | YASPIN | | | | | | | | | | | | | | H | H | H | H | H | H | H | H | H | H | H | | | | | | | E | E | E | | | | | | |
| β4 | SYMPRED | | | | | | | | | | | | | H | H | H | H | H | H | H | H | H | H | | | | | | | | | | | | | | | | | |
| α4PolyA | AA | H | V | E | T | **A** | **A** | **A** | **A** | **A** | **A** | **A** | **A** | R | A | H | A | E | E | R | *L* | L | K | K | *L* | F | S | G | Y | N | K | W | S | R | P | V | A | N | I | S |
| α4PolyA | GorV | | | | | | | | | | | | H | H | H | H | H | H | H | H | H | H | H | H | H | H | | | | | | | | | | | | | | |
| α4PolyA | Jpred | | | | | | | | | | | | | | | H | H | H | H | H | H | H | H | H | H | H | H | H | H | H | H | H | H | | | | | | | |
| α4PolyA | PROF | | | | | | | | | | | | | | H | H | H | H | H | H | H | H | H | H | H | H | H | H | H | H | H | H | | | | | | | | |
| α4PolyA | PSIPRED | | H | H | H | H | H | H | H | H | | | | H | H | H | H | H | H | H | H | H | H | H | H | H | H | | H | H | H | H | H | | | | | | | |
| α4PolyA | SSPRO | | | H | H | H | | | | | | | | H | H | H | H | H | H | H | H | H | H | H | H | H | H | | | | | | E | | | | | | | |
| α4PolyA | YASPIN | | H | H | H | H | H | H | H | H | | | | | | | H | H | H | H | H | H | H | H | H | H | H | H | | | | | | | | | | | | |
| α4PolyA | SYMPRED | | | H | H | H | | | | | | | | | H | H | H | H | H | H | H | H | H | H | H | H | H | | | | | | | | | | | | | |
| β4FLAG4 | AA | | R | V | A | N | **D** | **Y** | **K** | **D** | **D** | **D** | **D** | **K** | A | E | E | K | *L* | M | D | D | *L* | L | N | K | T | R | Y | N | N | L | I | R | P | A | T | S | S | S |
| β4FLAG4 | GorV | | | | | H | H | H | H | H | H | H | H | H | H | H | H | H | | | | | | | | | | | | | | | | | | | | | | |
| β4FLAG4 | Jpred | | | | | | | | | | | | | H | H | H | H | H | H | H | H | H | H | H | H | H | | | | | | H | H | | | | | | | |
| β4FLAG4 | PROF | | | | | | | | | | | H | H | H | H | H | H | H | H | H | H | H | H | | | | | H | H | H | | | | | | | | | | |
| β4FLAG4 | PSIPRED | | | | | | | | | | | H | H | H | H | H | H | H | H | H | H | H | H | H | H | H | H | H | H | H | H | H | | | | | | | | |
| β4FLAG4 | SSPRO | | | | H | H | H | H | H | H | H | H | H | | | | | | H | | | | | | | | | | | | | | | | | | | | | |
| β4FLAG4 | YASPIN | | | | | | | | | | | | H | H | H | H | H | H | H | H | H | H | H | H | | | | | | | | | | | | | | | | |
| β4FLAG4 | SYMPRED | | | | | | | | | | | H | H | H | H | H | H | H | H | H | H | H | H | | | | | | | | | | | | | | | | | |

Supplemental Figure 1. Sequences and predicted secondary structures for selected constructs. Predicted structures are shown for wild-type subunits (top 2 sets of data), that agree relatively well for all prediction programs we used. Two constructs that produced more divergent predictions are shown in the bottom 2 sets of data. The divergence suggests that the structure in this region of these constructs may not be well defined. As an estimate for the extent of agreement, we tabulated the number of programs that predicted a given residue would be in a helical region (coded as H in the prediction) in a given construct ("residue predicted number") and also tabulated the number of residues that at least one program predicted would be in a helical region ("predicted helical residues"). The sum of the (residue predicted numbers) divided by the (predicted helical residues) will range from 1 when there is no agreement among the predictions to a number equal to the number of programs used when there is total agreement (in our analysis 6). The values we observed for the 6 programs we used ranged from a low of 2.2 (β4V5) to a high of 4.6 (α4HA) with a mean of 3.5. For the 4 alignments shown in the Figure the values were 4.4 (α4), 4.1 (β4) 3.9 (α4PolyA) and 3.0 (β4Flag4). About equal fractions of residues were classified as helical by a single program (23%) or by all 6 programs (29%), with intermediate levels less frequent.
