# Supplementary material for: Introduced Amino Terminal Epitopes Can Reduce Surface Expression of Neuronal Nicotinic Receptors
Source: PLoS One. 2016 Mar 10;11(3):e0151071. doi: 10.1371/journal.pone.0151071 (PMC4786271; doi:10.1371/journal.pone.0151071)
Supplement: S2 Fig — (PDF) [file pone.0151071.s002.pdf]

Bracamontes et al

Supplemental figure 2

Construct																														
β4	R	V	A	N	A	E	E	K	*L*	M	D	D	*L*	L	N	K	T	R	Y	N	N	L	**I**	R	P	A	T	S	S	S
β4				H	H	H	H	H	H	H	H	H	H																	
β4 1stL	R	V	A	N	A	E	E	K	**P**	M	D	D	L	L	N	K	T	R	Y	N	N	L	**I**	R	P	A	T	S	S	S
β4 1stL					H	H	H	H	H	H	H	H	H																	
β4 2ndL	R	V	A	N	A	E	E	K	L	M	D	D	**P**	L	N	K	T	R	Y	N	N	L	I	R	P	A	T	S	S	S
β4 2ndL				H	H	H	H	H	H	H	H	H	H					H	H	H										

Supplemental Figure 2. Sequences and predicted secondary structures for 2 mutations in the α1 helix mentioned in the Discussion. The first sequence is wild type, followed by two constructs in which conserved leucine residues in the α1 helix are mutated to proline (Castillo et al. (2009) J Neurochem 108:1399-1409). The predicted structure is that produced by SYMPRED.
